# ﻿Two new species of *Deutereulophus* Schulz (Hymenoptera, Eulophidae) from China, with a key to Chinese species

**DOI:** 10.3897/zookeys.1114.86598

**Published:** 2022-07-20

**Authors:** Jun-Jie Fan, Cheng-De Li

**Affiliations:** 1 School of Forestry, Northeast Forestry University, Harbin, 150040, China Northeast Forestry University Harbin China

**Keywords:** Chalcidoidea, Eulophinae, *tennysoni* species-group, parasitoid, taxonomy

## Abstract

Two new species of *Deutereulophus* Schulz, *D.felix***sp. nov.** and *D.daguisiensis***sp. nov.**, are described from China. A key to species of *Deutereulophus* known from China is provided.

## ﻿Introduction

*Deutereulophus* Schulz is one of several small genera of the tribe Eulophini, subfamily Eulophinae (Hymenoptera: Eulophidae). Species of the genus are distributed in all zoogeographic regions, except for the Afrotropical region. Currently *Deutereulophus* contains 23 valid species (Noyes 2019): 10 species from the Australasian region (Girault 1913, 1915, 1922; Yoshimoto and Ishii 1965; Bouček 1988); seven species from the Oriental region (Zhu and Huang 2002a, 2002b; Ubaidillah 2003; Narendran et al. 2007; Narendran 2011); five species from the Nearctic region (Schauff 2000); three species from the Palearctic region (Erdös 1951; Zhu and Huang 2002a; Bae and Jung 2021); and two species from the Neotropical region (Ashmead 1904). Four of the species are known from China: *D.froudei* (Girault), *D.interruptus* Zhu & Huang, *D.marginatus* Zhu & Huang, and *D.tennysoni* (Girault) (Zhu and Huang 2002a, 2002b).

The genus *Deutereulophus* (as *Entedonomorpha* Girault) was subdivided into two species groups by Bouček (1988). This study describes two new species of the genus within the *tennysoni*-group and provides a key to all species occurring in China.

## ﻿Material and methods

All specimens were collected by sweeping or yellow-pan trapping, and were dissected and mounted in Canada balsam on slides following the method of Noyes (1982), or mounted on a card. Slide-mounted specimens were photographed with a digital CCD camera attached to an Olympus BX51 compound microscope. Specimens on cards were photographed with an Aosvi AO-HK830-5870T microscope. Measurements were made using the built-in software of Aosvi AO-HK830-5870T. The quality of these photos was improved by using Helicon Focus 7 and Adobe Photoshop 2020.

Terminology follows the Hymenoptera Anatomy Consortium (2022) for most body parts except the callus, which follows Gibson (1997). The following abbreviations are used:

**F1–3** flagellomeres 1–3;

**MV** marginal vein;

**OOL** minimum distance between a posterior ocellus and corresponding eye margin;

**PMV** postmarginal vein;

**POL** minimum distance between posterior ocelli;

**SMV** submarginal vein;

**STV** stigmal vein.

All type material is deposited in the insect collections at Northeast Forestry University (**NEFU**), Harbin, China.

## ﻿Taxonomy

### ﻿ Deutereulophus

Taxon classificationAnimaliaHymenopteraEulophidae

Schulz

5E640D32-3CF6-5ACB-AE2E-FE79868FC4EA

Eulophopteryx Ashmead, 1904: 341. Type species: Eulophopteryxchapadae Ashmead, by monotypy. Preoccupied by Eulophopteryx Möschler 1878: 684.
Deutereulophus Schulz, 1906: 146. Replacement name for EulophopteryxAshmead 1904.
Entedonomorpha Girault, 1913: 261. Type species: Entedonomorphatennysoni Girault, by original designation. Synonymised with Deutereulophus Schulz by LaSalle and Schauff 1992: 17.
Bryopezus Erdös, 1951: 171. Type species: Bryopezusbrevipennis Erdös, by monotypy. Synonymised with Deutereulophus Schulz by Burks 2012: 26.

#### Diagnosis.

Female antenna with funicle 3- or 4-segmented; clava 3- or 4-segmented; male antenna with funicle 4- or 5-segmented; occiput concave, usually with an occipital carina; pronotum with sides rounded or parallel, without a transverse carina along anterior part of pronotal collar; notauli complete; midlobe of mesoscutum with 2 pairs of setae; mesoscutellum with sublateral grooves that are converging posteriorly and meeting medially or not; propodeum with middle part high, convex; metasoma with distinct petiole.

### ﻿*Key to Chinese species of Deutereulophus* Schulz based on females

**Table:** 

1	Funicle 3-segmented (Figs 3, 7)	**2**
–	Funicle 4-segmented	***D.froudei* (Girault)**
2	Sublateral grooves on mesoscutellum converging and meeting posteriorly (Figs 5, 6)	**3**
–	Sublateral grooves on mesoscutellum not meeting posteriorly (Zhu and Huang 2002a: 355, fig. 5)	**5**
3	Propodeum with a raised triangular cup-shaped area anteromedially (Fig. 6)	***D.daguisiensis* sp. nov.**
–	Propodeum without a raised triangular cup-shaped area anteromedially	**4**
4	POL 2.8× OOL; metasoma mostly yellow with margins dark brown to black (Fig. 5)	***D.felix* sp. nov.**
–	POL 1.6× OOL (Zhu and Huang 2002a: 356, fig. 17); metasoma completely yellow	***D.marginatus* Zhu & Huang**
5	Metasoma metallic green	***D.interruptus* Zhu & Huang**
–	Metasoma lemon yellow	***D.tennysoni* (Girault)**

### ﻿*Deutereulophustennysoni* species-group

### ﻿ Deutereulophus felix sp. nov.

Taxon classificationAnimaliaHymenopteraEulophidae

E6387482-B406-5202-B61C-E41A684983C6

https://zoobank.org/7A880BD3-AB31-4506-A7BC-11EB5A3157F7

Figs 1–5

#### Type material.

***Holotype***, ♀ [NEFU; on card], China, Hunan Province, Chenzhou City, Yongxing County, Bianjiang Town, Pengjiawan Village, 23–25. VII. 2021, Shu-Chen Deng, by yellow-pan trapping. ***Paratypes***: 4♀ [2 ♀ on slide, 2 ♀on cards], same data as holotype.

#### Diagnosis.

Head and mesosoma black. Face strongly reticulate with large meshes. Antennal scrobes smooth. Vertex with scattered pits. Antenna yellow with scape pale yellow. Female funicle 3-segmented, clava 4-segmented. Mesoscutum strongly reticulate with large meshes. Sublateral grooves on mesoscutellum converging and meeting posteriorly. Legs mostly yellowish-white. Metasoma yellow with margins dark brown to black.

#### Description.

**Female.** Length 1.4 mm, fore wing length 1.0 mm. Head and mesosoma black. Eyes gray. Ocelli pale yellow. Antenna yellow with scape pale yellow. Mandibles dark brown. Petiole black. Metasoma yellow with margins dark brown to black. Legs mostly yellowish-white. Wings hyaline with veins brown.

**Figure 1–5.**
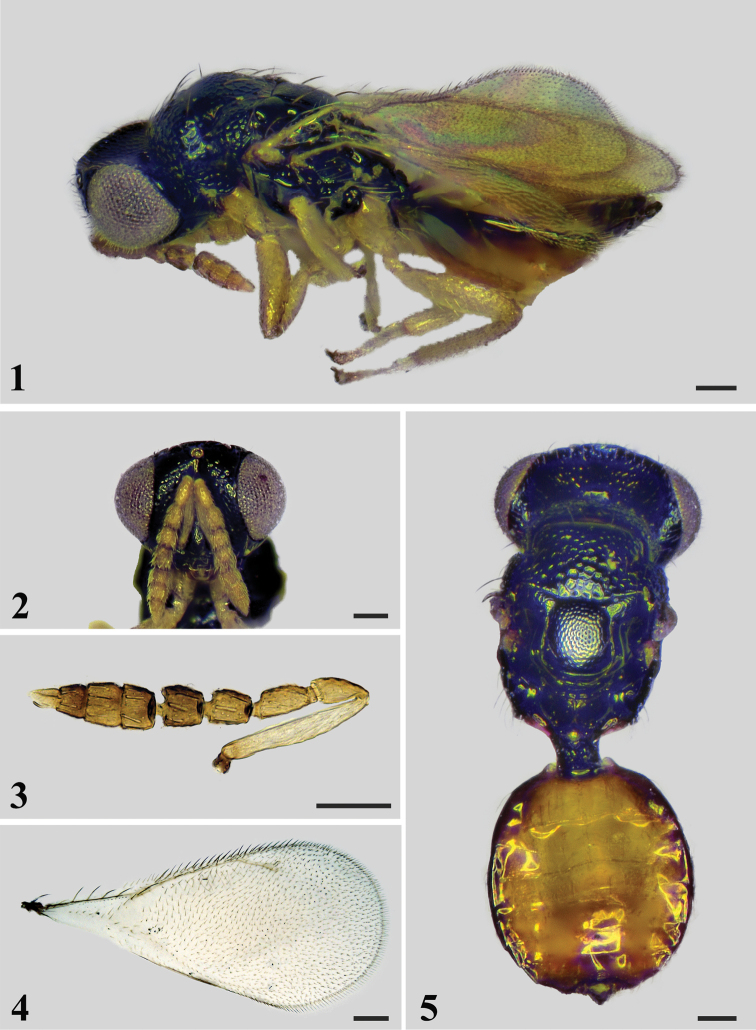
*Deutereulophusfelix* sp. nov., female, holotype **1** paratype (**2–5**) **1** habitus in lateral view **2** head in frontal view **3** antenna **4** fore wing **5** habitus in dorsal view. Scale bars: 100 μm.

**Head** (Fig. 2) 1.3× as wide as high in frontal view and 2.2× as wide as long in dorsal view. Face strongly reticulate with large meshes. Antennal scrobes smooth, reaching anterior ocellus. Vertex with scattered pits and short setae. Occiput strongly reticulate, occipital carina present. Eyes with extremely short and sparse setae. POL 2.8× OOL. Malar sulcus present, malar space 0.44× eye height. Mandible with one large tooth which includes three small teeth at its apex. Antenna (Figs 1, 3) with scape slender and cylindrical, 6.6× as long as wide; pedicel 2.0× as long as wide and scape 2.9× as long as pedicel; funicle 3-segmented, F1 2.1× as long as wide and almost as long as pedicel, F2 1.3× as long as wide, F3 1.1× as long as wide; clava 4-segmented, 2.8× as long as wide. Relative measurements (length: width): scape = 40: 6; pedicel = 14: 7; F1 = 15: 7; F2 = 12: 9; F3 = 12: 11; clava = 34: 12.

***Mesosoma*** (Figs 1, 5) 1.4× as long as wide. Pronotum rectangular, strongly reticulate with large meshes, covered with numerous setae and 2 considerably long setae posteromedially, without transverse carina along anterior margin of pronotal collar. Mesoscutum strongly reticulate with large meshes, midlobe of mesoscutum with 2 pairs of long setae. Axillae slightly advanced with faint reticulation. Mesoscutellum weakly reticulate, meshes of reticulation smaller and shallower than meshes on mesoscutum, with 2 pairs of long setae; sublateral grooves united posteriorly. Propodeum about 0.5× as long as length of mesoscutellum measured medially, smooth, median carina split and diverging posteriorly, plicae and paraspiracular carina present; spiracles separated from metanotum by a distance shorter than their own diameter; each propodeal callus with 9 setae.

***Wings*.** Fore wing (Fig. 4) 2.1× as long as wide. SMV with 5 setae on dorsal surface. Cubital vein straight at base. Speculum small, closed posteriorly. Relative measurements (length): SMV = 36; MV = 36; PMV = 12; STV = 16.

***Metasoma*** (Figs 1, 5) almost as long as mesosoma. Petiole longer than wide in dorsal view. Metasoma ovate, 1.1× as long as wide; first tergite 0.3× as long as length of metasoma. Ovipositor exserted beyond apex of metasoma.

**Male.** Unknown.

#### Host.

Unknown.

#### Distribution.

China (Hunan).

#### Etymology.

Named after the Latin adjective *felix*, meaning lucky.

#### Remarks.

The new species is similar to *Deutereulophusmarginatus*, and can be separated using the key given above.

### ﻿ Deutereulophus daguisiensis sp. nov.

Taxon classificationAnimaliaHymenopteraEulophidae

08C5DC71-9949-5063-A04A-0D42C97F314A

https://zoobank.org/02F37BE0-42A2-4F4D-B908-7AF3845F0280

Figs 6–9

#### Type material.

***Holotype***, ♀ [NEFU; on card], China, Hubei Province, Suizhou City, Daguisi National Forest Park, 12. VI. 2012, Guo-Hao Zu and Jiang Liu, by sweeping. ***Paratypes***: 1♀ [on slide], same data as holotype.

#### Diagnosis.

Head and mesosoma black. Vertex with scattered pits. Antennal scrobes smooth. Antenna yellowish-white with F3 dark brown, clava dark brown with apex yellowish-white. Female funicle 3-segmented, clava 4-segmented. Legs yellowish-white with procoxa and profemur dark brown. Face with raised reticulation. Metasoma dark brown. Mesoscutum strongly reticulate with large meshes. Sublateral grooves on mesoscutellum converging and meeting posteriorly. Propodeum with a raised triangular cup-shaped area anteromedially, median carina split and diverging posteriorly.

#### Description.

**Female.** Length 2.4 mm, fore wing length 1.7 mm. Head and mesosoma black. Eyes gray. Ocelli pale yellow. Antenna yellowish-white with F3 dark brown, clava dark brown with apex yellowish-white. Mandibles dark brown. Petiole black. Metasoma dark brown. Legs yellowish-white with procoxa and profemur dark brown. Wings hyaline with veins brown.

***Head*** (Fig. 8) 1.5× as wide as high in frontal view and about 2.3× as wide as long in dorsal view. Face with raised reticulation. Antennal scrobes smooth, reaching anterior ocellus. Vertex with scattered pits. Occiput with raised reticulation, occipital carina present. POL 2.3× OOL. Eyes bare. Malar sulcus present, malar space 0.41× eye height. Antenna (Fig. 7) with scape slender and cylindrical, 6.5× as long as wide; pedicel 1.8× as long as wide, and scape 3.7× as long as pedicel; funicle 3-segmented, F1 2.7× as long as wide and 1.7× as long as pedicel, F2 1.7× as long as wide, F3 1.3× as long as wide; clava 4-segmented, 3.0× as long as wide. Relative measurements (length: width): scape = 59: 9; pedicel = 16: 9; F1 = 27: 10; F2 = 20: 12; F3 = 18: 14; clava = 42: 14.

**Figure 6–9.**
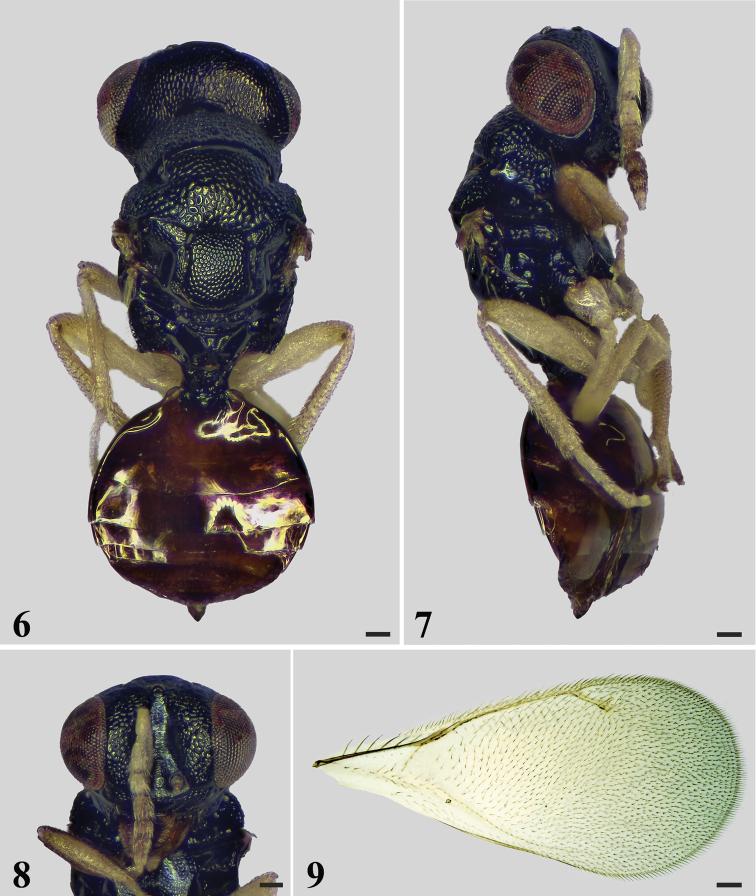
*Deutereulophusdaguisiensis* sp. nov., female, holotype **6** habitus in dorsal view **7** habitus in lateral view **8** head in frontal view **9** fore wing. Scale bars: 100 μm.

***Mesosoma*** (Fig. 6) 1.4× as long as wide. Pronotum rectangular with raised reticulation, covered with numerous setae, without transverse carina along anterior margin of pronotal collar. Mesoscutum strongly reticulate with large meshes. Axillae advanced anteriorly with faint reticulation. Mesoscutellum weakly reticulate, meshes of reticulation smaller and shallower than meshes on mesoscutum, with 2 pairs of long setae; sublateral grooves meeting posteriorly. Propodeum smooth; anteromedially with a raised triangular cup-shaped area; median carina split and diverging posteriorly; spiracles separated from metanotum by a distance shorter than their own diameter.

***Wings*.** Fore wing (Fig. 9) 2.4× as long as wide. SMV with 7 setae on dorsal surface. Cubital vein straight at base. Speculum small, closed posteriorly. Relative measurements (length): SMV = 29; MV = 32; PMV = 12; STV = 10.

***Metasoma*** (Figs 6, 7) almost as long as mesosoma. Petiole rugose, wider than long in dorsal view. Metasoma rounded, as long as wide; first tergite 0.4× as long as length of metasoma. Ovipositor exserted beyond apex of metasoma.

**Male.** Unknown.

#### Host.

Unknown.

#### Distribution.

China (Hubei).

#### Etymology.

Named after the type locality, the Daguisi National Forest Park in Hubei Province.

#### Remarks.

The new species is similar to *D.malabarensis* Narendran, but can be separated from it by the following combination of characters: antenna yellowish-white with F3 dark brown, clava dark brown with apex yellowish-white (antenna dark brown except pale scape in *malabarensis*); sublateral grooves on mesoscutellum converging and meeting posteriorly (not meeting posteriorly in *malabarensis*); propodeum with a raised triangular cup-shaped area anteromedially (without a raised triangular cup-shaped area in *malabarensis*).

## Supplementary Material

XML Treatment for Deutereulophus

XML Treatment for Deutereulophus felix

XML Treatment for Deutereulophus daguisiensis
